# Development of an efficient chromatin immunoprecipitation method to investigate protein-DNA interaction in oleaginous castor bean seeds

**DOI:** 10.1371/journal.pone.0197126

**Published:** 2018-05-08

**Authors:** Mohammad Enamul Haque, Bing Han, Bin Wang, Yue Wang, Aizhong Liu

**Affiliations:** 1 Key Laboratory of Economic Plants and Biotechnology, Yunnan Key Laboratory for Wild Plant Resources, Kunming Institute of Botany, Chinese Academy of Sciences, Kunming, China; 2 University of Chinese Academy of Sciences, Beijing, China; 3 Key Laboratory for Forest Resources Conservation and Utilization in the Southwest Mountains of China, Ministry of Education, Southwest Forestry University, Kunming, China; Saint George's University, UNITED KINGDOM

## Abstract

Chromatin immunoprecipitation (ChIP) is usually a reliable technique to find the binding sites of a transcription factor. In the current study, we developed a suitable ChIP method using developing castor bean seeds. A castor bean seed with large and persistent endosperm contains high amounts of storage lipids (ca. 50–60%) and is often considered as a model material to studying seed biology. In oleaginous seeds, due to the rich oils which could seriously affect immunoprecipitation and DNA isolation, it is often difficult to carry out a successful ChIP experiment. Thus, the development of an efficient ChIP method for oleaginous seeds is required. In this study, we modified different steps, including tissue preparation for cross-linking, chromatin washing, sonication and immunoprecipitation of other existing methods. As exemplified by the targeted gene identification of a master regulator WRI1, which regulates fatty acid biosynthesis, we found that the improved ChIP method worked well. We analyzed percentage input and fold changes of the ChIPed DNA. We also made successful ChIP-seq libraries using this method. This method provides a technical support not only for use on castor bean seeds; it might be used equally to analyze protein-DNA interaction *in vivo* in other oleaginous seeds.

## Introduction

Identification of binding sites of a given transcription factor with its target gene is an important step for gene regulatory network analysis. Protein-DNA interaction mediates diverse biological processes such as transcription, replication, DNA housekeeping, recombination and gene silencing. Many experimental methods such as chromatin immunoprecipitation (ChIP) [1–3], Bacterial one-hybrid assay [4] and yeast one-hybrid assay [5] have been broadly used to determine the protein-DNA interactions. Particularly, ChIP combined with deep sequencing (ChIP-seq) is an efficient and reliable method to find target genes and location of the DNA binding site for a specific transcription factor or protein associated with chromatin, and has become a critical strategy to dissect the Protein-DNA interaction *in vivo* using native cells or tissues. ChIP-chip is another technique generally used to detect protein-DNA association *in vivo* [2, 6, 7]. Due to several limitations of ChIP-chip (e.g., lower resolution, and dependant on hybridization and high coverage affinity for non-repetitive regions), ChIP-seq is becoming a more powerful technique with advantages such as nucleotide specific resolution (which is the greatest advantage over ChIP-chip), since due to the absence of the hybridization step ChIP-seq is free from noise, showing a distinct peak which is biologically meaningful; moreover, it has no limitation on genome coverage [8]. At present, although many protocols have been developed to carry out the ChIP experiment in different plant species, most of these protocols are designed for soft tissues like leaves [9–11], stems and seedlings [9, 12, 13], or for some special tissues such as Arabidopsis mesophyll protoplasts [14], wood-forming stem-differentiating xylem (SDX) tissues [15], and unicellular green algae [16]. However, little research has been done on the ChIP experiment using seeds as starting materials [17, 18]. Seeds are highly specific and special organs or tissues. It is important to dissect the molecular mechanisms underlying regulation and storage reservoir accumulation during seed development. Usually, it is difficult to carry out a successful ChIP experiment in seeds due to the high amount of storage compounds such as polysaccharides, lipids and some secondary metabolites. These storage compounds could seriously affect different steps of the experiment, especially immunoprecipitation, resulting in low amount of DNA after isolation. For oilseed crops, rich lipids within tissues also cause a difficulty in isolating quality genomic DNA and RNA from seeds [19, 20]. In particular, as the demand for plant oil increases for both food and industrial applications, it is necessary to develop and improve oilseed crops through the enhancement of oil content and improvement of fatty acid composition for human health or for industrial applications [21]. Thus, it is an immediate need to develop an efficient and reliable ChIP method to dissect the molecular regulation process of seed development (in particular for oilseeds).

The castor bean (*Ricinus communis* L.) is one of the most important non-edible oilseed crops and its seed oil is widely used in industry, such as for the production of lubricating oil and biodiesel [22, 23]. Its seed contains more than 60% storage lipids (in the form of triacylglycerols) and is rich in ricinoleic acids. Unlike Arabidopsis and other dicotyledonous oilseed crops such as rapeseed, sunflower, groundnut and soybean (their oils are accumulated in cotyledons and their endosperms are ephemeral in the early stage of seed development), castor bean seed oils and other storage reservoirs (such as storage proteins and polysaccharides) are accumulated in the endosperm and its endosperm is persistent through seed development. Thus, the castor bean is often considered as a model dicotyledonous plant for studying the molecular regulation of seed development and storage material biosynthesis in the field of seed biology [24, 25]. Due to the lack of an efficient and suitable ChIP method, studies on dissecting the molecular mechanism of seed development and storage material biosynthesis have been largely restricted for the castor bean.

Previously, we tried to use different ChIP methods [2, 10, 12] in the experiment of castor bean seed ChIP-seq—commonly used on leaves, shoots and other soft tissues and could not obtain enough targeted ChIP-ed DNA from developing seeds for further analysis due to the rich presence of lipids and polysaccharides. Here, we modified the common ChIP method by optimizing several experimental steps, and have developed a suitable and efficient method to dissect the molecular mechanism of seed development and oil biosynthesis in castor bean seeds. We have resolved some solution formulations and some difficulties such as sonication, washing of the nuclear pellet, immunoprecipitation, washing of beads-immune complexes, etc. As the master regulator WRI1 of oil biosynthesis [26] exemplified, we have demonstrated a successful and detailed protocol to conduct the ChIP experiment using developing castor bean seeds. We trust this method to not only provide an optimized experimental procedure for conducting ChIP in castor bean seeds, but also be suitable for use in investigating the protein-DNA interaction in other oleaginous seed crops.

## Materials and methods

### Plant materials

In this study, we used castor bean variety ZB107 as plant materials (kindly provided by the Zibo Academy of Agriculture Sciences, Shandong, China). Seeds were cultivated in the experimental field of the Kunming Institute of Botany, Chinese Academy of Sciences, during the months of April to October, 2016, when we maintained the plant’s growth under natural conditions. During flowering, we performed hand pollination to prevent cross pollination and to maintain proper records from the day after pollination (DAP). Endosperms were isolated from the seed samples collected from 7, 15, 25, 35 and 45 DAP respectively, and snap-frozen immediately to extract RNA.

### Total RNA purification and complementary DNA synthesis

Total RNA was purified from the collected endosperm samples using TRIzol (Invitrogen, Carlsbad, CA), followed by RNeasy Mini Kit (Qiagen, Valencia, CA), according to the manufacturer’s instructions. Then we checked the concentration and quality of total RNA by NANODROP-2000 spectrophotometer (Thermo Scientific) and by 1.5% agarose gel as well. Complementary DNA for different stages of seed samples were synthesized from 1 μg of total RNA by transcript one-step gDNA removal and cDNA synthesis supermix (TIANGEN, Beijing, China), according to the manufacturer’s instructions, and then the cDNA samples were stored at -20°C until use.

### Lipid analysis

Total lipid was extracted according to Xu et al. and Zhang et al. [27, 28]. At first, the seed samples were collected from five developmental stages and dried in a vacuum incubator. The dried samples for three replications were measured accurately. The seed tissues were ground with a mortar and pestle to fine powder using liquid nitrogen, transferred into 10 ml glass tubes, and then added 2 ml of hexane:isopropanol (3:2, v/v) and mixed well. To collect the supernatant, the mixtures were centrifuged at 5000 g for 5 min. After that, total lipids were extracted by drying the supernatants in a vacuum incubator at 50°C to remove the dissolved hexane phase, and determining gravimetrically.

### Primer design

For primer design, we obtained the CDS sequences (for Rc*WRI1* and Rc*EF1α*) and promoter sequences (for Rc*BCCP2*, Rc*EAR1* and Rc*DGAT1*) from Phytozome v12.1 through query of the specific gene’s protein sequence from Arabidopsis. Prime Primer 5.0 (Premier, Palo Alto, CA, USA) was used to design primers with amplicon sizes between 100–180 bp. For ChIP qPCR, we designed primers from the promoter sequences of Rc*BCCP2* and Rc*EAR1* containing AW box. All the primers are listed in S1 Table.

### Quantitative real-time PCR analysis

Quantitative real-time PCR was performed by the CFX96 system (Bio-Rad, Hercules, CA, USA) to analyze tissue specific expression of the WRI1 transcription factor and ChIP-ed DNA enrichment. For ChIP qPCR, the ChIP-ed DNA, 10% input DNA and negative control DNA (no Ab sample) were diluted three times with deionized water. PCR amplifications were carried out in 20 μL reaction mixture containing the following: 0.5 uL of DNA template, 8.7 uL of deionized water, 10 uL of TransStart Tip Green qPCR SuperMix (TransGen Biotech, Beijing, China), and 0.8 μL of forward and reverse primers. The two-step PCR program consisted of initiation at 95°C for 30 s, then denaturation at 95°C for 15 s, annealing at 60°C for 30 s, and extension at 95°C for 10 s with 40 cycles. The melting curve was obtained from 65°C to 95°C, at an increment of 0.5°C every 5 s. The Rc*EF1α* was used as an internal control for gene expression normalization of Rc*WRI1* and the reaction was performed three times. In the case of ChIP qPCR, the enrichments were analyzed in two biological and three technical replications.

### Western blotting

For western blotting, proteins were purified from 0.5 g of young leaves and 1 g of seeds of 35 DAP castor bean according to Fido et al. [29] and quantified by the Bradford method [30]. Proteins were also purified from decrosslinked samples of IP and mock control. In this case, a 20 μl solution from each of the samples was taken and precipitated by 3 volumes of acetone at -20°C for 30 min. The tubes were centrifuged at 12000 rpm for 5 min to pellet the proteins, and then the pellets were washed with 70% ethanol, dried briefly and dissolved with 20 μl of 2x Laemmli Buffer. Proteins were also purified from another set of IP and mock control samples. Before being run on SDS-PAGE, 2x Laemmli Buffer was also added to 40 μg of crude protein extracts from the leaf and seed samples, and the protein samples were denatured at 95°C for 10 min and run on SDS-PAGE. After that the proteins were transferred on PVDF membrane using Trans-Blot^®^ SD Semi-Dry Transfer Cell (BIO-RAD). The membrane was blocked in 5% BSA in TBST at room temperature for 1 hour, and then added primary antibody and incubated at room temperature for 3 hours with gentle shaking. The membrane was rinsed 3 times for ten minutes with TBST. Then it was incubated with the secondary antibody for 2 hours at room temperature and washed with TBST for three times in the same way as previous washing steps. Finally the image was developed by ECL Prime Western Blotting Detection Reagent (GE Healthcare Life Sciences) using MicroChemi 4.2 (DNR Bio-imaging Systems, ISRAEL) according to the manufacturer’s instructions. In this study, we used rabbit anti-WRI1 antibody (Willget Biotech Co., China) as the primary antibody and goat anti-rabbit IgG/HRP (Biosharp, China) or Anti-Rabbit IgG HRP (Rabbit TrueBlot) (Rockland Immunochemicals Inc.) as the secondary antibody.

### ChIP experiment

**Fig 1:**
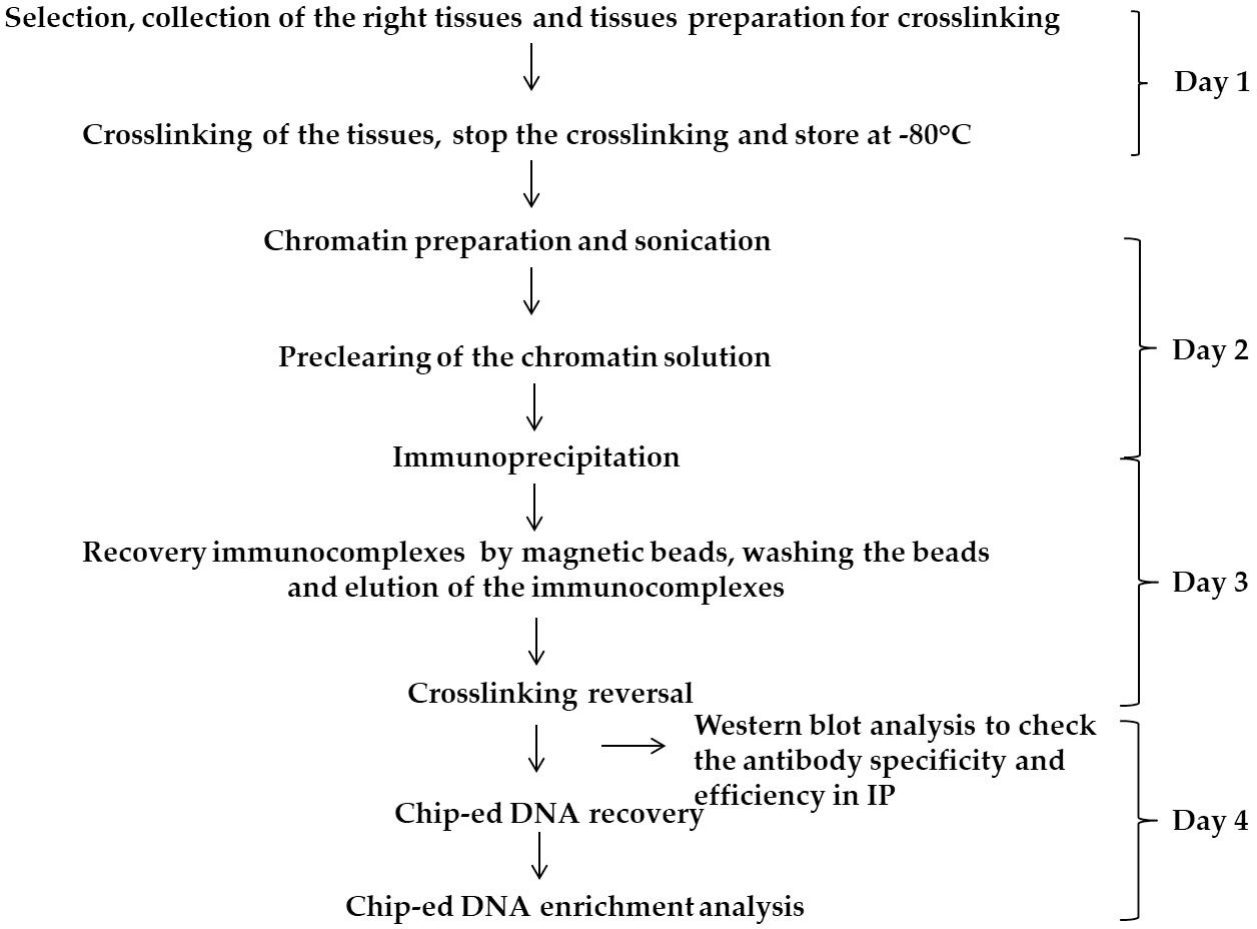
Diagrammatic representation of the modified ChIP experiment that was used in the castor bean oilseed.

The overall procedure of our ChIP experiment is diagrammatically shown in Fig 1. Based on the existing ChIP experiment methods [2, 10, 12] which are usually used in soft tissues such as leafs and seedlings, we modified the solution formulations and some critical steps. First, crosslinking is a critical step that largely affects the final acquisition of targeted DNA fragments [3, 10]. The seed tissues were cut very thin to increase the penetration of crosslinking agents to the tissues. Before crosslinking, we washed the dissection tissues quickly with fixation buffer without formaldehyde to remove most lipids and other debris. Second, to further clean the lipids and other debris the nuclear pellets were washed several times using extraction buffer 2 and 3 during chromatin extraction. Third, after sonication, we added centrifugation of the chromatin solution several times to further remove debris. Fourth, during the washing of beads we extended the time and number of washes of the beads with washing buffers. For preparing fixation buffers we applied 1x PBS (pH 7.4) with 0.1 M sucrose and 1x Protease inhibitor, as PBS and sucrose induce crosslinking in the hard lipid tissues. For extraction buffer 1, 2 and 3, compared with the previous buffer systems described by Kaufmann et al. [2] we added 1 mM PMSF and 1 mM EDTA, and increased the concentration of 2-methyl 2, 4-pentanediol up to 1.5 M to easily remove lipids from the nuclear extract and precipitate proteins completely during several centrifugations. In addition, we added 0.1% Trition X-100 in buffer 3 to help extensively wash lipids off the nuclear pellet. For the magnetic beads washing buffers and elution buffers we adopted the buffer combination described by Ricardi et al. [10] and Bowler et al. [12]. The detailed procedure of the ChIP experiment is given in S1 File.

### ChIP qPCR analysis and performed ChIP-seq

ChIP qPCR data were analyzed by percentage input [31] and fold enrichment using the 2^(-ΔΔCt)^ method [32]. Input samples were used as positive controls and no antibody-treated samples were used as negative controls. Rc*DGAT1* was used as a negative control locus. After checking the enrichment, the DNA samples were sequenced by Illumina HiSeq 2000 on the BGI platform (BGI, Shenzhen, China).

## Results and discussion

### Selection of tissues and preparation for crosslink

**Fig 2:**
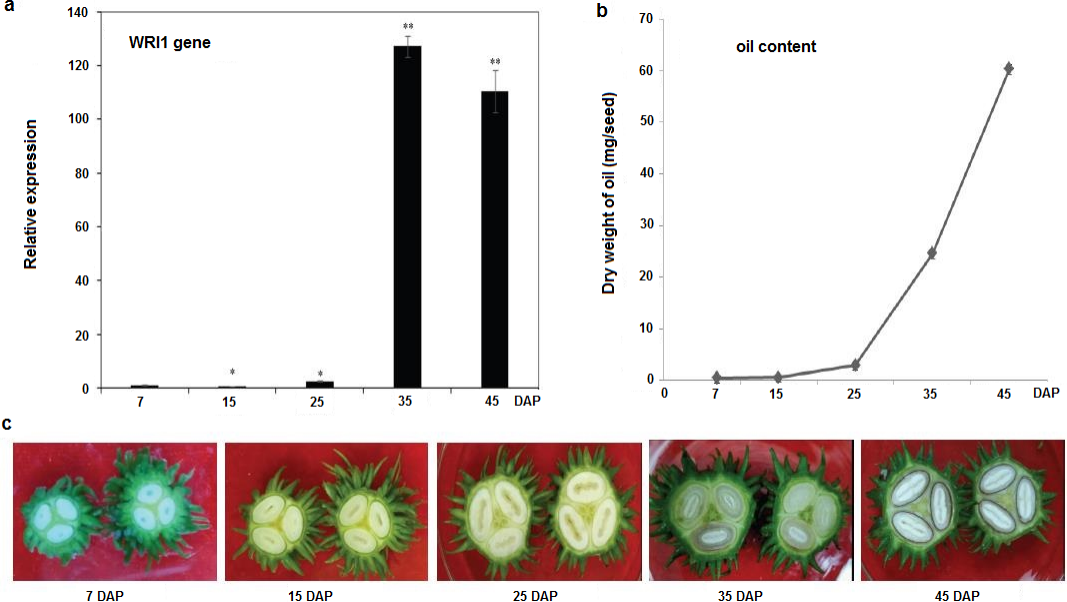
Relative expression of the WRI1 transcription factor, oil contents and different developmental stages of the castor bean seed. a qPCR was performed to analyze the relative expression of the WRI1 transcription factor in different seed stages. **b** and **c** Oil contents and morphology of the different developmental stages of the seeds. Tissues from different developmental stages (7, 15, 25, 35, 45 DAP) of the seeds were used to check the expression levels, analyze oil content and study seed morphology. DAP indicates day after pollination, error bars indicate the SEM. Statistical significance was carried out by pair-sample t-test. Different asterisks indicate different significant levels: one asterisk (*) for P < 0.05 and two asterisks (**) for P < 0.01. qPCR data were normalized to Rc*EF1α*, a housekeeping gene.

As most of the transcription factors are usually tissue specific in their expression, it is inevitable that a right tissue is selected where the desired transcription factor is abundant for its regulation. It is also a key step for the success of the ChIP experiment, or the recovery quality and quantity of the targeted DNA over the control. Here, to find out the right seed developmental stage for master transcription factor WRI1 [26, 33], which regulates many fatty acid biosynthetic genes for lipid accumulation during seed development, we applied a real-time qPCR technique to investigate the expression of WRI1 at different stages of seed development, including 7, 15, 25, 35 and 45 DAPs. As shown in Fig 2A, WRI1 is expressed highly at 35 DAP, implying that WRI1 is rapidly expressed between 25 to 35 DAP. Correspondingly, the oil content is rapidly accumulated between 25 to 35 DAP (Fig 2B). Meanwhile, we can observe visible endosperm development at 35 DAP (Fig 2C). Thus, we collected the seed samples from 35 DAP. Similarly, storage lipids accumulation is concentrated from the mid to late phase of seed development in most oilseeds [27, 34–36], thus sampling the developing seed tissues at the mid-phase of seed development might be critical for most oilseed crops, to dissect the target genes of a given transcription factor functionally involved in regulating lipids (or other storage materials) accumulation during seed development.

The crosslinking reaction is a critical step in making a successful ChIP experiment. According to our experience, for the seed tissues, it is necessary to cut the seeds into very thin pieces prior to crosslinking. In this experiment, we made homogenous tissues for generating uniform and complete crosslinking reactions, because the dissection tissues could be easily infiltrated. Although there have been several reports on the optimization of crosslinking using different concentrations of formaldehyde in different types of tissues [3, 11, 37], we here optimized the sonication conditions using a standard concentration (1%) of formaldehyde [2, 11] and obtained relatively ideal results.

### Chromatin processing and immunoprecipitation

**Fig 3:**
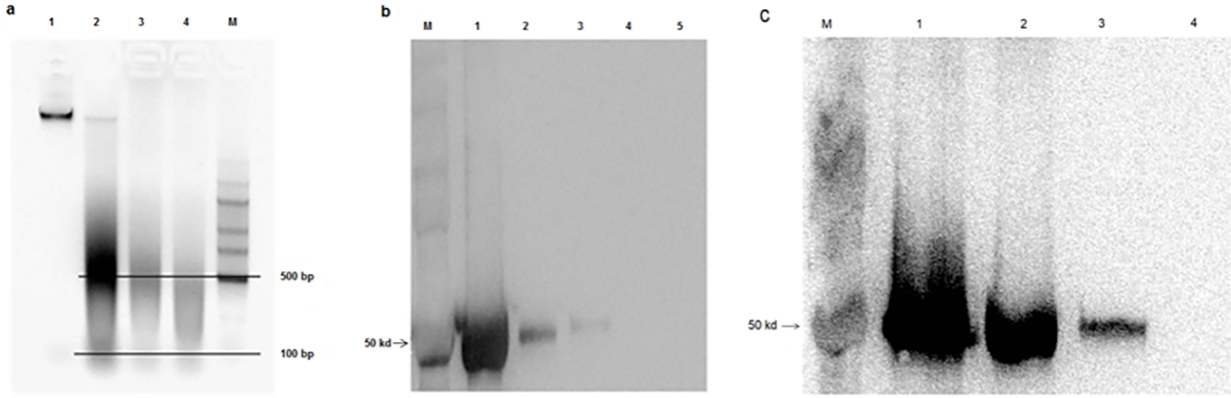
Sonication efficiency and antibody specificity. **a** Chromatin samples were fragmented at 20 sec ON/30 sec OFF using Bioruptor® Pico. Samples were decrosslinked, DNA was purified by phenol:chloroform:isoamyl alcohol extraction and loaded on 1.5% agarose gel. Lane 1: non-sonicated; lane 2: sonicated 7 times; lane 3: sonicated 15 times; lane 4: sonicated 20 times; lane M: 5000 bp DNA marker. Scales indicate the DNA fragments to be between 100–500 bp. **b** Western blot figure showing lane M: protein molecular weight marker; lane 1: crude protein extract (40 μg); lane 2: IP in sample 1; lane 3: IP in sample 2; lane 4: mock control in sample 1; lane 5: mock control in sample 2. Arrow indicates the protein marker band 50 KD. **c** Western blot showing the lane M protein weight marker; lane 1: crude protein extract (40 μg) from endosperms of 35 DAP castor bean seeds; lane 2: IP sample; lane 3: IP sample (20 μl from the IP sample used for lane 2); lane 4: mock control sample. Arrow indicates the protein marker band 50 KD.

Chromatin processing, including chromatin washing and fragmentation, is always considered to be a key step in obtaining a good result for all ChIP experiments. In this method, we optimized several critical steps (of washing) to clear the supernatant. These improved steps enhance the binding efficiency of the antibody to the beads and reduce more background during IP. However, this procedure has a drawback as it causes a bit of loss in some chromatins. For a study of protein binding sites genome-wide, it is important to shear the chromatin efficiently in desired lengths. Different ChIP methods have mentioned their sonication conditions differently; in most of the cases the fragment size ranges from 200–2000 bp [15, 16, 31]. However, the sonication conditions depend on the tissue, chromatin sample volume and sample concentration. In our experiment, we tried to optimize the shearing conditions using Bioruptor® Pico (DIAGENODE, BELGIUM) with a combination of 20 sec ON/30 sec OFF for several times, and obtained maximum fragments in the range of 100–500 bp in 20 times on agarose gel (Fig 3A). We have also checked the DNA size of the IP sample on the Agilent 2100 Bioanalyzer, and found the pick range to be between 100–500 bp (see S1 Fig). Thus, we recommend dissolving the chromatin pellet with lysis buffer according to the primary tissue taken for the experiment and the efficiency of sonication. Lipids make the chromatin solution more viscous, which generates heat in the solution, reduces fragmentation efficiency and denatures protein during sonication. Generally smaller fragments are expected in a ChIP experiment; however, DNA fragments within 100–500 bp are able to meet the purposes of the ChIP experiment.

We have mentioned another key step of the ChIP experiment in lipid seeds, which is washing the beads following IP. We washed the beads twice with each of the washing buffers carefully (see methods), to thoroughly remove lipids and other seed-derivatives which were attached to the beads during IP. However, excessive washing could cause the loss of DNA and this has generally happened if the tissues are not crosslinked properly. After the final wash of the beads, we performed decrosslinking over-night at 65°C [37] to recover the maximum amount of DNA. We purified protein from 20 μl of decrosslinked solution of IP and mock control samples, that was run on the SDS-PAGE alone with 40 μg of crude protein extract (from 35 DAP seed stage), following western blot analysis to check the efficiency of IP and antibody specificity. We got the protein bands at the position of 50 KD on the membrane (Fig 3B), lane 1 for crude protein sample, lanes 2 and 3 for IP of sample 1 and sample 2, and lanes 4 and 5 for the mock controls of sample 1 and 2, respectively. However we confused with the protein size at the position of 50 KD as the Ig heavy chain has also the similar molecular weight. We used goat anti-rabbit IgG/HRP secondary antibody which could bind to Ig heavy chain also, even the membrane was washed extensively before and after antibodies treatment which may reduce the presence of nonspecific binding. To confirm specific protein for WRI1, we further checked the IP following western blot of another set of IP and mock control samples along with a crude protein extract using anti-Rabbit IgG HRP (Rabbit TrueBlot) secondary antibody (which is unable to recognize denatured immunoglobulins) and we found the protein band at the position of 50 KD for WRI1 (Fig 3C), suggesting that our ChIP method work efficiently. For lane 3 in Fig 3C, proteins of 20 μl IP’s sample was loaded which was taken from the IP sample loaded on lane 2, as we have mentioned to perform western blot using small amount of IP sample after decrosslink. Before the ChIP experiment we also checked the antibody specificity by western blot using the crude protein extract from the endosperms of 35 DAP seeds and young leaves (see S2 Fig), and we found the antibody to be specific.

### DNA enrichment analysis in ChIP samples by qPCR

**Fig 4:**
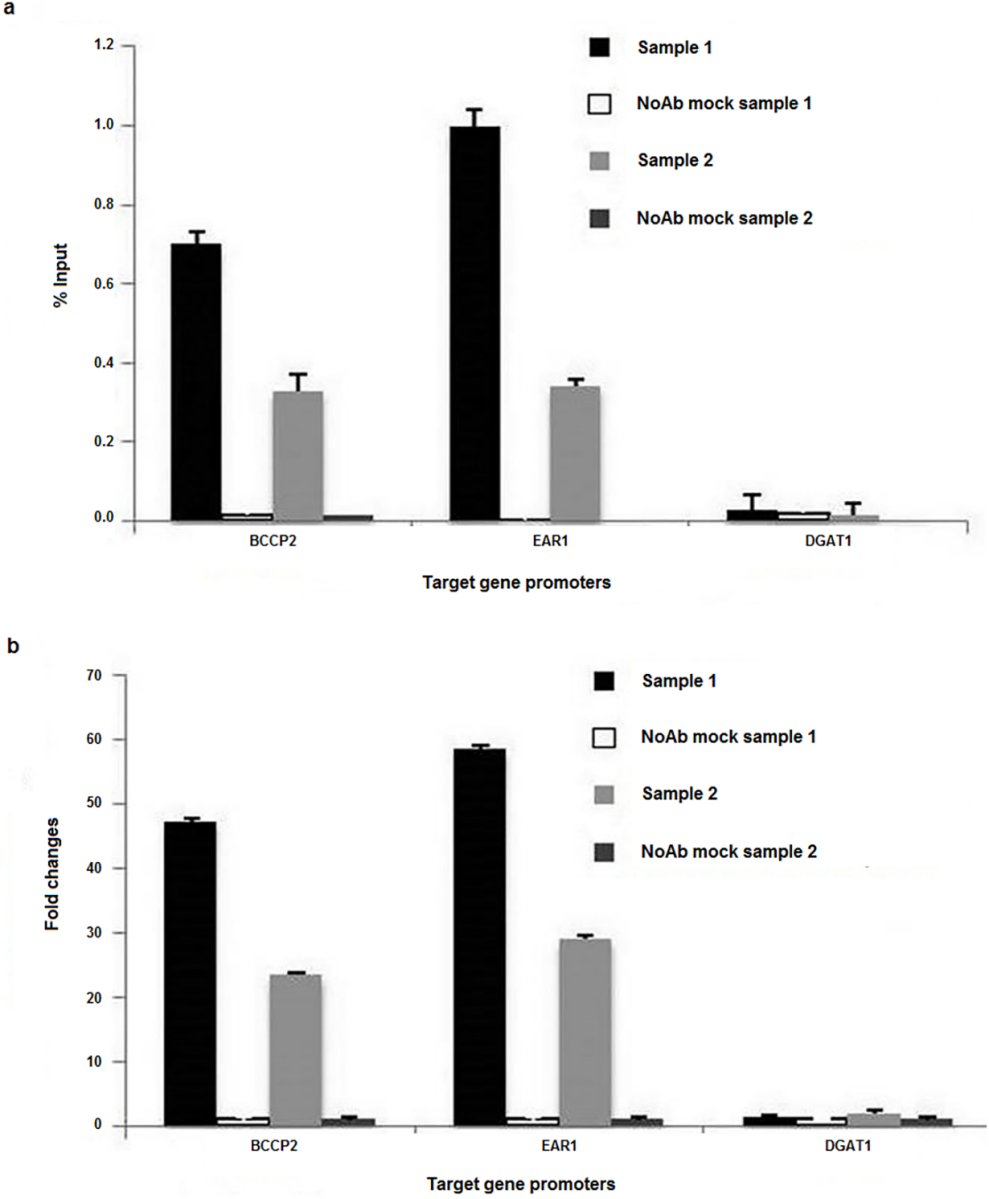
Percentage input and fold changes analysis. **a** percentage input and **b** fold changes of the target regions in the promoters of Rc*BCCP2*, Rc*EAR1* and Rc*DGAT1*. Rc*DGAT1* was used as a negative control locus. The ChIP results were obtained from two biological and three technical replications. Error bars indicate the SEM values.

Several techniques have been used to validate the ChIP-ed DNA [11]; however, we performed qPCR followed by analysis of input percentage and fold changes using ChIP-ed DNA, input DNA and mock control DNA. We designed the primers for qPCR to contain AW box, the binding site of the WRI1 transcription factor [38]. Then we analyzed the enrichment of the binding site in the promoter of Rc*BCCP2* and Rc*EAR1*, the two key enzymes in fatty acid biosynthesis [39–41]. We also analyzed the enrichment of binding loci in the promoter of Rc*DGAT1* as a negative control, the non-target gene of the WRI1 transcription factor [38]; however, it is a key-enzyme gene responsible for TAG assembly in the Kennedy pathway [42]. We performed qPCR of two samples using three technical replications. Percentage input and fold changes were normalized to the input DNA (10%) for each of the promoter regions. In the case of percentage input, the Rc*BCCP2* region showed input percentage values of 0.7 and 0.32 in sample 1 and sample 2 respectively, whereas the Rc*EAR1* region showed values of 0.99 and 0.33 for sample 1 and sample 2 respectively. In the case of the Rc*DGAT1* region, % input values in ChIP-ed DNA were not significant and more or less similar to the no antibody mock control for both of the samples (Fig 4A). We also analyzed the fold changes in each of the samples: more than 20-fold for both of the target regions. On the other hand, the non-target region (Rc*DGAT1*) showed no fold changes over the control in both of the samples (Fig 4B), suggesting that our developed ChIP method was efficient and suitable for conducting protein-DNA interaction in the developing castor bean seeds.

### Quality analysis of ChIP-sequencing libraries

To test whether the quality of the obtained ChIP libraries is sufficient for ChIP-seq analysis high throughput DNA sequencing was applied. After sequencing, the raw reads were mapped to the reference genome of the castor bean using SOAP2 (version 2.21t) [43], to analyze uniquely mapped reads and quality of the reads. From the analyzed data, we found the mapped rates for sample 1, sample 2 and input-sample 1 to be 92.58%, 89.84% and 93.87% respectively. Meanwhile, the uniquely mapped rates for sample 1, sample 2 and input-sample 1 were 57.79%, 59.36% and 65.27% respectively (Table 1), suggesting that the quality of the libraries was good for further ChIP-seq analysis.

**Table 1:** Clean reads, mapped reads and uniquely mapped reads of sequencing data.

Sample name	Clean Reads Number	Clean Rate (%)	Mapped Reads	Mapped Rate (%)	Uniquely Mapped Reads	Uniquely Mapped Rate (%)
Sample 1	21,912,297	99.87	20,286,833	92.58	12,663,834	57.79
Sample 2	21,926,008	99.93	19,699,186	89.84	13,015,131	59.36
Input sample 1	21,774,009	99.24	20,438,626	93.87	14,211,823	65.27

### Conclusions

Due to the presence of lipids and other metabolic compounds, it is difficult to perform ChIP on seed tissues using other methods mentioned for soft tissues like leaves, stems, seedlings, etc. In this study, we provide a simple and optimized chromatin immunoprecipitation method for lipid seeds to analyze protein-DNA interaction *in vivo*. We optimized some important steps such as tissue preparation for crosslinking, sonication and immunoprecipitation to minimize the problems generated by lipids and other metabolic compounds and we also optimized the solution formulations. We have shown sound results, as exemplified by the investigation of the targeted gene’s enrichment of the WRI1 transcription factor in lipid castor bean seeds. This method provides a technical support not only for use in castor bean seeds, but also for equal use in the global genetic analysis of other oleaginous seeds.

## Supporting information

S1 File The detailed procedure of the ChIP experiment. (PDF)

S1 Fig DNA fragments checked by Agilent 2100 Bioanalyzer. Figure showing maximum fragments ranges between 100–500 bp. Peaks at 35 and 10380 showing lower and upper markers.
(TIF)

S2 Fig Western blot analysis to check anti-WRI1 antibody specificity. Figure showing the lane M protein weight marker; lane 1 and 2: crude protein extract from young leaves; lane 3 and 4: crude protein extract from endosperms of 35 DAP castor been seeds. In all cases, 40 μg of crude protein extracts were run on gel. Arrow indicates the protein marker band 50 KD.
(TIF)

S1 Table Primer sequences used for qPCR analysis. (PDF)

## Abbreviations

ChIP: chormatin immunoprecipitation
RT: room temperature
TAGs: triacylglycerols
WRI1: Wrinkled1
SDS: sodium dodecyl sulfate
EDTA: ethylenediaminetetraacetic acid
NP-40: Nonidet P-40
PMSF: phenylmethylsulfonyl fluoride
IP: immunoprecipitation
TE: tris-ethylenediaminetetraacetic acid
EP tube: Eppendorf tube
PAGE: polyacrylamide gel electrophoresis
ChIP-qPCR: chormatin immunoprecipitation-quantitative PCR
Rc: *Ricinus communis*
BCCP2: biotin carboxyl carrier protein 2
EAR1: enoyl-acyl carrier protein reductase1
DGAT1: diglyceride acyltransferase 1
