# Supplementary material for: Development of an efficient chromatin immunoprecipitation method to investigate protein-DNA interaction in oleaginous castor bean seeds
Source: PLoS One. 2018 May 8;13(5):e0197126. doi: 10.1371/journal.pone.0197126 (PMC5940234; doi:10.1371/journal.pone.0197126)
Supplement: S1 File — (PDF) [file pone.0197126.s001.pdf]

The detailed protocol developed for investigating protein-DNA interaction in oleaginous seeds of castor bean

Day 1: Tissue collection and crosslinking

1. At first the fruits were collected from the suitable developmental stage of castor bean. Then seeds were isolated and endosperms were extracted. The endosperms were cut in to small and thin pieces by using single-edged blade and kept on ice in a petri dish.
2. 3 g of dissecting tissues were taken into a 50 ml falcon tube and washed away lipids with 20 ml of fixation buffer without formaldehyde.
3. Then 37 ml fixation buffer was added to the tube, mixed several times and crosslinked under the vacuum incubator for a total of 15 min. First time the vacuum incubation was done for 3 min then repeated twice (6 min vacuum/release/mix).
4. Crosslinking was stopped by adding 2 M glycine (2.5 ml) and applied vacuum again for 5 min.
5. Tissues were washed 3 times with fixation buffer (without formaldehyde) and dried them quickly by paper towels.
6. After then the materials were wrapped with aluminum foil, freezed in liquid nitrogen and kept at -80°C for up to 3 months.

Day 2: Chromatin extraction and shearing and Immunoprecipitation

7. Tissues were ground with pre-cooled mortar pestle and liquid nitrogen to fine powder. Then transferred to pre-cooled 40 ml extraction buffer 1 in a 50 ml falcon tube.

8. The tube was vortexed for a few seconds and kept on ice for 15 min. During this time, the tube was mixed several times to dissolve the powder properly.
9. Then the mixture was filtered twice through miracloth and collected the supernatant into new pre-cooled 50 ml falcon tube. We filtered the mixture first time through one layer and second time two layers of miracloth.
10. Nuclear pellet was extracted by centrifugation the solution at 1500g for 20 min at 4°C.
11. After centrifugation, the supernatant was discarded and dissolved the pellet with 5 ml of extraction buffer 2 followed by centrifugation at 1000g for 10 min at 4°C.
12. Step 11 was repeated for four times to wash nuclei.
13. Again the nuclear pellet was washed with 5 ml of extraction buffer 3 for three times.
14. Then the pellet was resuspended in 0.6 ml of lysis buffer by pipetting with a cut-off 1 ml tip and saved 10 µL of resuspended chromatin at -20°C to check on gel.
15. The chromatin solution was taken into 8 x 0.65 ml of sonication tubes where each of the tubes contained 100 µL of the solution.
16. Then we perform sonication using Bioruptor® Pico with following condition: 20 cycles, 20 sec ON/30 sec OFF at 4°C in sonication bath to generate the fragment size between 100-500 bp. After 10 cycles, solutions were mixed by pipetting to get better sonication efficiency. To check the degree of sonication on 1.5 % agarose gel, 10 µL of fragmented chromatins from 7, 15 and from final cycle were taken and kept at -20°C.
17. The chromatin solutions were then combined in to a new 1.5 ml eppendorf tube and centrifuged at 14000g for 10 min.

18. After centrifugation, the supernatant was transferred in to a new 1.5 ml tube carefully. At this stage, we found three distinct layers in the tube, the first layer was lipid layer, second layer was chromatin solution and the third was cellular debris. To avoid lipid, we carefully transferred the chromatin solution in to a new tube.
19. Again we centrifuged the nuclear solution twice at 12000g for 10 min to rescue the supernatant from lipid as possible and transferred in to new EP tubes.
20. Then we diluted the supernatant with equal amount of IP buffer and again centrifuged at the same condition of step 19.
21. At this time, we prepared magnetic protein A+G beads by taking 3x 40 µL of beads into 1.5 ml EP tube and washed three times with 1 ml of IP buffer. Each time, the solution was kept on magnetic strand, hold for 1 min on ice and the liquid was removed carefully without beads.
22. 40 µL of the beads was added to the chromatin solution by a cut-off pipette tip and incubated the chromatin solution on a slowly rotating wheel for 2 h at 4°C to preclear the solution.
23. After incubation, the chromatin solution was separated by magnetic strand for 1 min on ice.
24. Then the supernatant was taken into a new tube and 10 % chromatin solution was saved for input control to check the enrichment of the IP sample by PCR.
25. The supernatant was divided into two tubes equally, one for IP and another for mock control. Then added 1-2 µg of anti-WRI1 antibody to the IP sample and the mock

control was prepared without antibody. The chromatin solutions were incubated for overnight at 4°C on a slowly rotating wheel.

Day 3: Recovery immunocomplexes and Reverse crosslinking

26. To recover the immunocomplexes, we added 40 µL of beads from step 21 to the IP and mock tubes by a cut-off pipette tip and incubated at least 6 hours at 4°C on a gently rotating wheel.
27. Then rescued the beads from IP and mock samples on magnetic strand and carefully removed the supernatant from each of the tubes without disturbing beads.
28. After then, the beads of IP and mock control samples were washed twice with each of the washing buffers 1, 2 and 3 by maintaining the first step wash quickly for 20 second and the second step for 10 min on a rotating wheel at 4°C. In between wash, the beads were separated on the magnetic strand for 1 min on ice, and removed the buffer thoroughly.
29. The beads were washed twice with TE buffer for 10 min on rotating wheel at 4°C and collected beads on the magnetic strand.
30. For elution, we added 250 µL of elution buffer to the IP and control beads and incubated at 65°C for 15 min. The tubes were vortexed shortly for several times during incubation and immunocomplexes were collected on the magnetic strand at RT. The elution of the immunocomplexes was repeated once more and combined the two elutants.

31. After then, 20 µL of 5 M NaCl was added to the elutants of IP and mock control and inverted the tubes several times to mix well. At the same time, the tubes of steps 14, 16 and

24 from -20°C were removed and centrifuged at 12000 g for 5 min at 4°C. The supernatants were transferred into new tubes and added elution buffer up to 500 µL, and then in each of the tubes we added 20 µL of 5 M NaCl. Then we did decrosslinking of the solutions by incubating the tubes at 65°C for overnight.

Day 4: DNA recovery and analysis

32. In this step, we checked IP efficiency and antibody specificity by rescuing proteins from decrosslinked solutions of IP and mock control samples. Then proteins were precipitated by 3-4 volumes of acetone precipitation. The tubes were centrifuged at 12000 rpm for 5 min to pellet the proteins, and then pellets were washed with 70 % ethanol. We dried the protein pellets briefly at RT and dissolved with 20 µL of 2 x laemmli buffer. Then the protein samples were denatured at 95°C for 10 min and run on SDS PAGE along with a crude protein extract of seeds. Then the proteins were transferred on a PVDF membrane followed by the steps of western blot. In this case, we used anti-WRI1 antibody as primary antibody from rabbit source and goat anti-rabbit IgG/HRP as secondary antibody. We further confirmed the WRI1 specificity by using another IP and a mock control samples in western blot, where Anti-Rabbit IgG HRP (Rabbit TrueBlot) (Rockland Immunochemicals Inc.) was used as secondary antibody.
33. We also continued the step from 31 by adding 2 µL (10 mg/ml) DNase free RNase A to the decrosslinked solution of all tubes and incubated at 37°C for 1 h.

34. To digest protein, 1 µL of 20 mg/ml proteinase K alone with 10 µL of 0.5 M EDTA (pH 8.0) and 20 µL of 1 M Tris.HCl (pH 6.5) were added to each of the tubes and incubated for 1 h at 45°C in a water bath.
35. Then DNA were recovered by equal amount of phenol:chloroform:isoamyl alcohol (25:24:1) treatment, followed by chloroform:isoamyl alcohol (24:1) treatment. In both of the cases, the tubes were centrifuged at 12000 rpm for 10 min at RT.
36. The purified supernatants were transferred into new tubes and DNA were precipitated by 1 µL of 20 mg/ml glycogen, 1/10 volume of 3 M sodium acetate and two volumes of absolute ethanol for overnight at -20 °C.
37. After then, the tubes were centrifuged at 14000 g for 15 min at 4°C and the pellets were washed with 1 ml of 70 % ethanol and again centrifuged at 7500 g for 5 min at 4 °C.
38. The pellets were dried briefly at RT and dissolved the pellets of IP, input and mock control with 20 µL of nuclease free water. At the same time, DNA pellets of other tubes dissolved with 30 µL of nuclease free water and checked the degree of sonication on 1.5 % agarose gel. We used Qubit® 2.0 Fluorometer (Invitrogen) to measure the concentration of DNA of IP, input and mock samples and stored at -20 °C.
39. Finally we performed qPCR to check the target DNA enrichment in the IP and mock control samples.
