# Supplementary figures and images for: Development of an efficient chromatin immunoprecipitation method to investigate protein-DNA interaction in oleaginous castor bean seeds
Source: PLoS One. 2018 May 8;13(5):e0197126. doi: 10.1371/journal.pone.0197126 (PMC5940234; doi:10.1371/journal.pone.0197126)

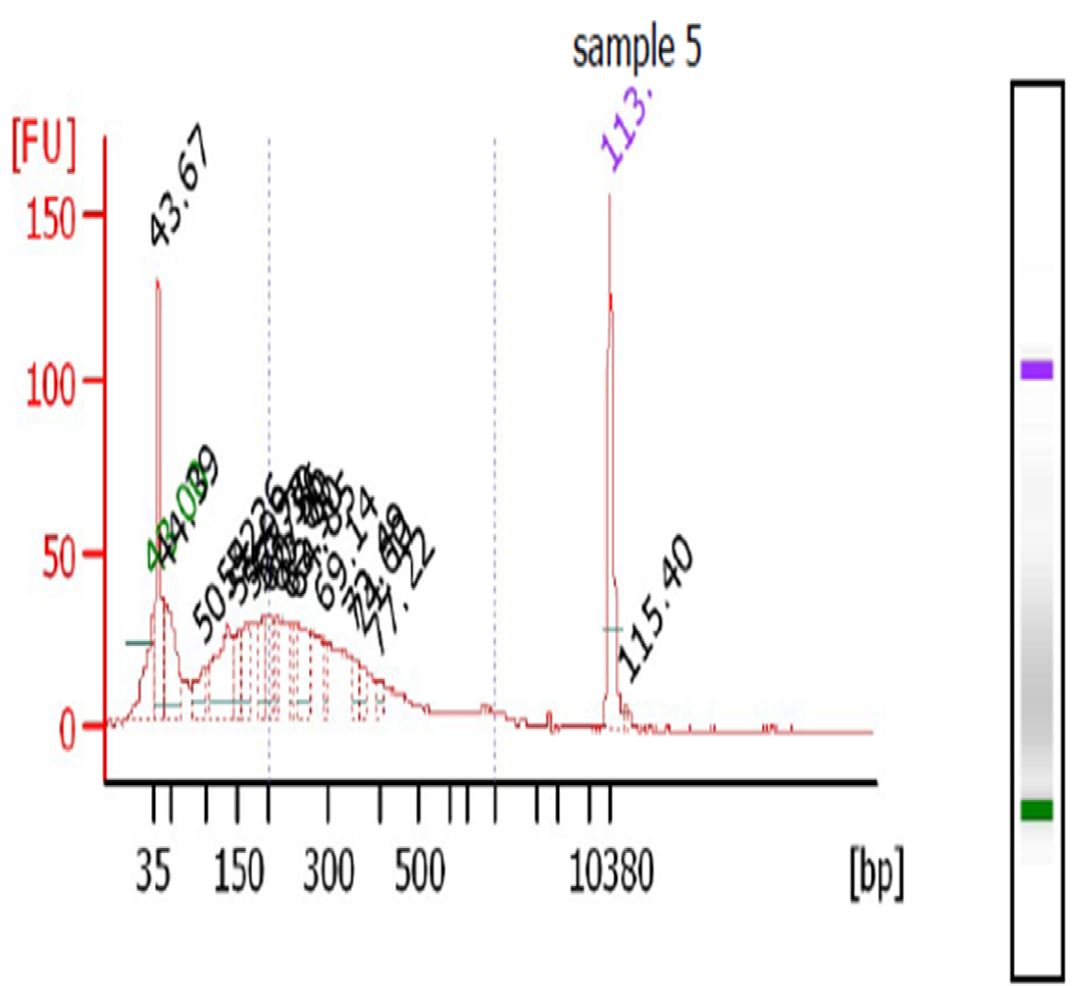

Supplement: S1 Fig — Figure showing maximum fragments ranges between 100–500 bp. Peaks at 35 and 10380 showing lower and upper markers. (TIF) [file pone.0197126.s002.tif]

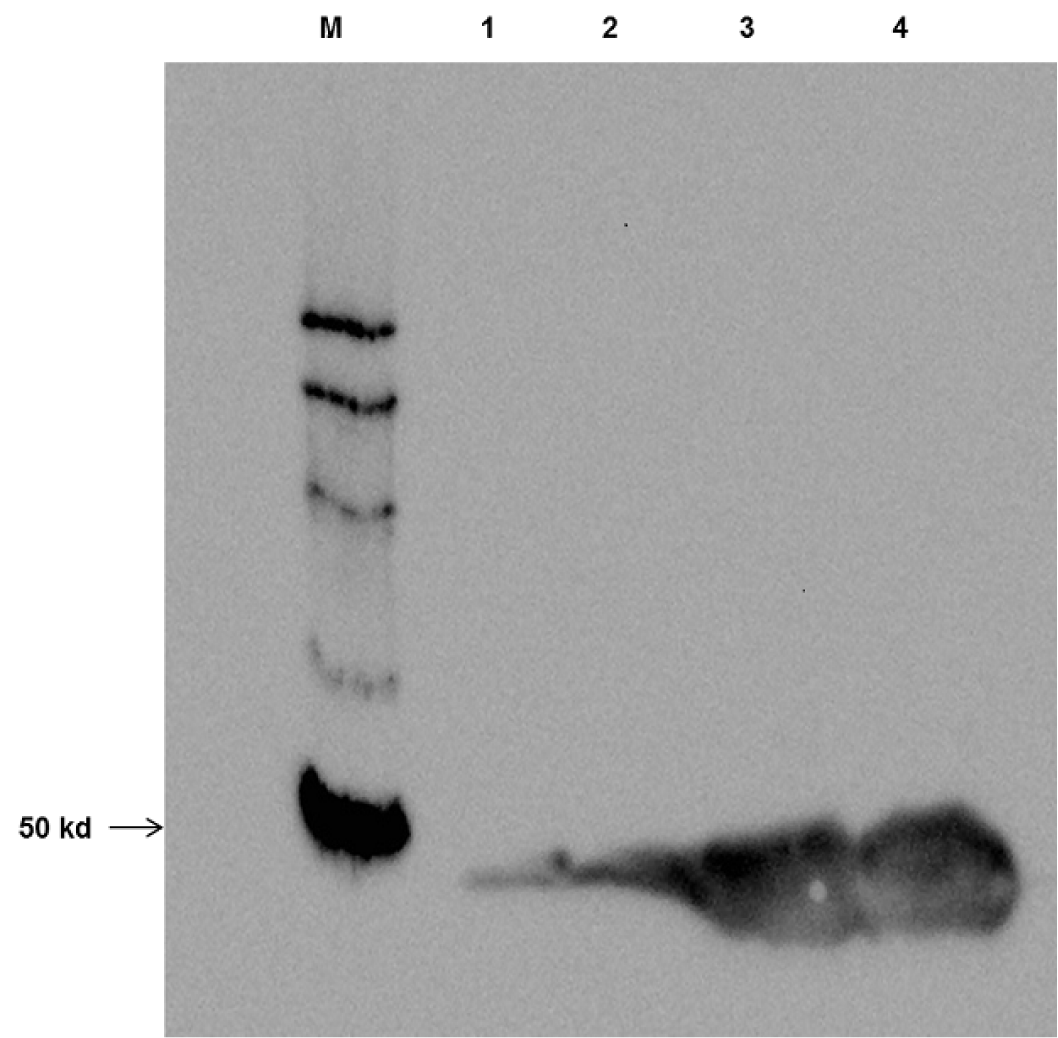

Supplement: S2 Fig — Figure showing the lane M protein weight marker; lane 1 and 2: crude protein extract from young leaves; lane 3 and 4: crude protein extract from endosperms of 35 DAP castor been seeds. In all cases, 40 μg of crude protein extracts were run on gel. Arrow indicates the protein marker band 50 KD. (TIF) [file pone.0197126.s003.tif]
