# Supplementary material for: Development of an efficient chromatin immunoprecipitation method to investigate protein-DNA interaction in oleaginous castor bean seeds
Source: PLoS One. 2018 May 8;13(5):e0197126. doi: 10.1371/journal.pone.0197126 (PMC5940234; doi:10.1371/journal.pone.0197126)
Supplement: S1 Table — (PDF) [file pone.0197126.s004.pdf]

S1 Table. Primer sequences used for qPCR analysis

Primer name	Sequences
Rc*BCCP2* F	CAGACGCCCCATTCTCTTC
Rc*BCCP2* R	CGGAAAAAGGGTGGCGATG
Rc*EAR1* F	CCACCTGTTTAGCCTCGG
Rc*EAR1* R	GGTGCTTCGAGAAGAGGAG
Rc*DGAT1* F	CTTCTTAGCGTGTCTTTGTC
Rc*DGAT1* R	CCAAGAGTTTCTGGCGTTTC
Rc*WRI1* F	TTACCACAAGAACAGGACACAATC
Rc*WRI1* R	AGAATCCAAGCAAAAACTCCAG
Rc*EF1α* F	TCAAGTATGCTTGGGTGCTG
Rc*EF1α* R	GGTCTCGAATTTCCACAAGG
